# Validity of an algorithm to identify cardiovascular deaths from administrative health records: a multi-database population-based cohort study

**DOI:** 10.1186/s12913-021-06762-0

**Published:** 2021-07-31

**Authors:** Lisa M. Lix, Shamsia Sobhan, Audray St-Jean, Jean-Marc Daigle, Anat Fisher, Oriana H. Y. Yu, Sophie Dell’Aniello, Nianping Hu, Shawn C. Bugden, Baiju R. Shah, Paul E. Ronksley, Silvia Alessi-Severini, Antonios Douros, Pierre Ernst, Kristian B. Filion

**Affiliations:** 1 Department of Community Health Sciences, University of Manitoba, Winnipeg, Manitoba Canada; 2 Center for Clinical Epidemiology, Lady Davis Institute, Jewish General Hospital, Montreal, Quebec, Canada; 3 Institut national d’excellence en santé et en services sociaux (INESSS), Quebec City, Quebec, Canada; 4 Department of Anesthesiology, Pharmacology and Therapeutics, University of British Columbia, Vancouver, British Columbia Canada; 5 Division of Endocrinology and Metabolism, Jewish General Hospital, McGill University, Montreal, Quebec, Canada; 6 The Health Quality Council, Saskatoon, Saskatchewan Canada; 7 College of Pharmacy, Rady Faculty of Health Sciences, University of Manitoba, Winnipeg, Manitoba Canada; 8 School of Pharmacy, Memorial University of Newfoundland, St John’s, Newfoundland and Labrador Canada; 9 ICES, Toronto, Ontario Canada; 10 Division of Endocrinology and Metabolism, Sunnybrook Health Sciences Centre, Toronto, Ontario Canada; 11 Department of Medicine, University of Toronto, Toronto, Ontario Canada; 12 Department of Community Health Sciences, Cumming School of Medicine, University of Calgary, Calgary, Alberta Canada; 13 Manitoba Centre for Health Policy, Rady Faculty of Health Sciences, University of Manitoba, Winnipeg, Manitoba Canada; 14 Departments of Medicine and of Epidemiology, Biostatistics, and Occupational Health, McGill University, Montreal, Quebec, Canada; 15 Institute of Clinical Pharmacology and Toxicology, Charité-Universitätsmedizin Berlin, Berlin, Germany

**Keywords:** Accuracy, Cause-specific mortality, Death certificates, Hospital records, Physician claims, Validation

## Abstract

**Background:**

Cardiovascular death is a common outcome in population-based studies about new healthcare interventions or treatments, such as new prescription medications. Vital statistics registration systems are often the preferred source of information about cause-specific mortality because they capture verified information about the deceased, but they may not always be accessible for linkage with other sources of population-based data. We assessed the validity of an algorithm applied to administrative health records for identifying cardiovascular deaths in population-based data.

**Methods:**

Administrative health records were from an existing multi-database cohort study about sodium-glucose cotransporter-2 (SGLT2) inhibitors, a new class of antidiabetic medications. Data were from 2013 to 2018 for five Canadian provinces (Alberta, British Columbia, Manitoba, Ontario, Quebec) and the United Kingdom (UK) Clinical Practice Research Datalink (CPRD). The cardiovascular mortality algorithm was based on in-hospital cardiovascular deaths identified from diagnosis codes and select out-of-hospital deaths. Sensitivity, specificity, and positive and negative predictive values (PPV, NPV) were calculated for the cardiovascular mortality algorithm using vital statistics registrations as the reference standard. Overall and stratified estimates and 95% confidence intervals (CIs) were computed; the latter were produced by site, location of death, sex, and age.

**Results:**

The cohort included 20,607 individuals (58.3% male; 77.2% ≥70 years). When compared to vital statistics registrations, the cardiovascular mortality algorithm had overall sensitivity of 64.8% (95% CI 63.6, 66.0); site-specific estimates ranged from 54.8 to 87.3%. Overall specificity was 74.9% (95% CI 74.1, 75.6) and overall PPV was 54.5% (95% CI 53.7, 55.3), while site-specific PPV ranged from 33.9 to 72.8%. The cardiovascular mortality algorithm had sensitivity of 57.1% (95% CI 55.4, 58.8) for in-hospital deaths and 72.3% (95% CI 70.8, 73.9) for out-of-hospital deaths; specificity was 88.8% (95% CI 88.1, 89.5) for in-hospital deaths and 58.5% (95% CI 57.3, 59.7) for out-of-hospital deaths.

**Conclusions:**

A cardiovascular mortality algorithm applied to administrative health records had moderate validity when compared to vital statistics data. Substantial variation existed across study sites representing different geographic locations and two healthcare systems. These variations may reflect different diagnostic coding practices and healthcare utilization patterns.

**Supplementary Information:**

The online version contains supplementary material available at 10.1186/s12913-021-06762-0.

## Background

Cardiovascular death is a cause-specific outcome of interest in many studies about the comparative effectiveness of new healthcare interventions. For example, studies about the safety and effectiveness of new prescription medications compared with existing medications frequently use both all-cause and cause-specific death as endpoints [1, 2]. When studies that include cause-specific mortality as an outcome are conducted using population-based data, vital statistics registration systems are often the preferred source of information about cause-specific mortality because they capture verified information about the deceased, the circumstances of death, and the direct antecedent and underlying cause(s) of death [3]. However, there can be challenges associated with using vital statistics registrations for population-based comparative effectiveness studies. The data may not be sufficiently timely for investigations of new interventions, such as new medications that have recently come to market, because the process required for verification of cause of death may be lengthy [4]. In addition, routine linkage of vital statistics registrations to other population-based administrative data may not be possible in all jurisdictions [5, 6], in part due to legislation governing data access [7].

Routinely-collected, population-based administrative health data, including hospital records and physician visit records, represent an alternative source to identify specific causes of death [8]. Administrative health data are potentially advantageous because in many jurisdictions, they are relatively straightforward to access, and processes have been established to link multiple sources of administrative data while ensuring that health privacy legislation requirements are met [9]. However, given that administrative data are captured for purposes of health system management and healthcare provider remuneration and not for identifying specific causes of death, their validity for the latter purpose has been questioned [10]. There are few studies that have examined the accuracy of administrative health data for investigating specific causes of death [10], particularly across multiple jurisdictions. A recent systematic review about sources of bias in drug safety and effectiveness studies conducted using population-based routinely-collected data emphasized the importance of validation studies to identify potential sources of bias and strategies to address these sources when measuring study exposures and outcomes [11].

The aim of our study was to assess the validity of an algorithm applied to administrative health records in multiple jurisdictions for identifying cardiovascular deaths. We used vital statistics registrations as the reference standard to validate the cardiovascular mortality algorithm.

## Methods

### Data sources

Data were from an existing multi-database retrospective cohort study conducted by the Canadian Network for Observational Drug Effect Studies (CNODES) [12], a pan-Canadian network that examines questions of drug safety and effectiveness at the request of government stakeholders. This cohort study investigated the safety and effectiveness of sodium-glucose cotransporter-2 (SGLT2) inhibitors, a new class of antidiabetic medications, compared to dipeptidyl peptidase-4 (DPP-4) inhibitors [13–16]. Databases from five Canadian provinces (Alberta, British Columbia, Manitoba, Ontario, and Quebec) and the United Kingdom (UK) Clinical Practice Research Datalink (CPRD) were used. The study period was from 2013 to 2018.

In each Canadian province, study data included vital statistics registrations, health insurance registrations, physician billing claims, hospitalization records, emergency department (ED) visit records (not available in Manitoba), and prescription drug dispensation records. These data sources can be linked at the individual level using anonymized personal health numbers. Vital statistics registrations capture official records of births, stillbirths, deaths, and marriages. In death records, the underlying cause of death is recorded using the World Health Organization’s International Statistical Classification of Diseases and Related Problems (ICD), 10th revision (i.e., ICD-10) [3]. The registration of deaths is a legal requirement in all Canadian provinces and as such, reporting is virtually complete; under-reporting may occur as a result of late or incomplete registration, but non-registration or over-reporting is unlikely [3]. Health insurance registration files capture start and end dates of health insurance coverage, including the date of loss of coverage due to death or migration; demographic and residence location information is also maintained in these files. Physician billing claims contain information about ambulatory services provided by specialists and general practitioners, including the type of service, date of service, and at least one diagnosis code associated with the reason for the service (in Quebec, some claims are missing a diagnosis code, although the overall completion rate is in excess of 88%); the latter are recorded using the 8th (Ontario only) and 9th revisions of ICD (i.e., ICD-8 and ICD-9) [17]. Hospitalization records contain information for each patient during the period of the hospital stay, including up to 25 diagnoses codes recorded using ICD-10-CA (i.e., enhanced Canadian revision). Prescription drug claims capture medications dispensed by community pharmacies; in-hospital medication dispensations are not included. ED visit records contain information about visits to hospital-based EDs, including the date of the visit, chief complaint (i.e., reason for the visit), and diagnosis codes (where available).

Study data were also obtained from the CPRD, a large UK primary care database containing medical information documented by primary care physicians for approximately 15 million patients enrolled in over 700 general practices [18]. The data are regularly reviewed and considered to be valid and of high quality [19–21]; they capture patient demographics, medical history, prescribed medications, and clinical measures, but do not capture emergency department (ED) visits. CPRD data were linked to the Hospital Episode Statistics (HES) database; this linkage is available for general practices in England that have consented to the linkage. The HES contain hospitalization information, including diagnoses recorded using ICD-10 codes. CPRD data were also linked to national death registrations from the Office of National Statistics (ONS); this linkage is available for general practitioners in England who have consented to the linkage. The underlying cause of death is recorded in registrations using ICD-10 codes.

### Study cohort

The cohort has been described in detail elsewhere [13–16]. Briefly, the cohort for the initial multi-database study included patients who received a prescription for a SGLT2 inhibitor or a DPP-4 inhibitor. The dispensation date (prescription date for CPRD) for either medication had to occur on or after the date of the first dispensation or prescription of a SGLT2 inhibitor for each site and on or before June 30, 2018. Cohort entry was the date of the first SGLT2 or DPP-4 inhibitor dispensation or prescription in this study period. Cohort exit was the date of censoring due to discontinuation of the study drug, death, end of healthcare coverage, or end of the study period. The initial study cohort excluded individuals less than 66 years of age in Ontario, 19 years in Alberta, and 18 years in British Columbia and Manitoba and in the CPRD. In Quebec, the initial cohort was restricted to individuals who were greater than 65 years, or who were receiving social assistance, or who did not have access to a private insurance plan. These exclusions were based on drug data availability in the sites. Additional exclusions from the initial study cohort were due to missing sex, date inconsistencies, no follow-up (i.e., cohort exit date less than or equal to cohort entry date), SGLT2 and DPP-4 inhibitor prescriptions on the same day after the cohort entry date, or less than 365 days of health insurance coverage prior to the cohort entry date.

We constructed our validation cohort from this initial study cohort for those sites where linkage of administrative health records and vital statistics registrations (death registrations from ONS in CPRD) was possible and for those years for which these registration data were available (see Table 1 for available data at each site). The validation cohort excluded individuals who were alive, based on health insurance coverage information in the Canadian provinces and no recorded date of death in the CPRD data, as of June 30, 2018. We subsequently excluded individuals who were missing a date of death, as well as individuals for whom the difference in dates of death recorded in administrative health records and vital statistics registrations was greater than 60 days; the latter was an indicator of potential data quality issues.

**Table 1:** Start and end dates of study period at each site for validation cohort creation

Site	Start and End Dates
Alberta	June 6, 2014 – December 31, 2016
British Columbia	June 3, 2014 – June 30, 2018
Manitoba	June 9, 2014 – December 31, 2017
Ontario	July 29, 2015 – December 31, 2016
Quebec	September 4, 2014 – December 31, 2016
Clinical Practice Research Datalink	February 4, 2013 – December 31, 2017

**Note:** Study period is limited to the years for which both vital statistics registrations and administrative health records were available at each site

### Outcome measure

The outcome of cardiovascular death in administrative health records used the following algorithm: (a) in-hospital death with a cardiovascular disease diagnosis in the primary/most responsible diagnosis position, or (b) out-of-hospital death (including death in an ED) without documentation of cancer in the 365 days prior to and including the date of death and without documentation of trauma in the 30 days prior to and including the date of death. A significant proportion of all cardiovascular-related deaths are known to occur outside of hospital [22–24]. We searched hospitalization records, ED visit records, and physician billing claims in provincial data, and all CPRD and HES records for documentation of cancer or trauma diagnoses for out-of-hospital deaths.

The list of relevant diagnosis codes to identify in-hospital cardiovascular deaths is provided in Table 2 [25]. For out-of-hospital cardiovascular deaths, the cancer diagnosis codes included ICD-9-CM 140 to 172 and 174 to 209 and ICD-10-CA C00 to C43 and C45 to C97, and the trauma-related diagnosis codes included ICD-9-CM 800 to 999 and E000 to E999 and ICD-10-CA S00 to T98 and V01 to Y98.

**Table 2:** ICD-10 diagnosis codes for cardiovascular disease

ICD-10 Codes	Code Description
I00-I02	Acute rheumatic fever
I05-I09	Chronic rheumatic heart diseases
I10-I15	Hypertensive diseases
I20-I25	Ischaemic heart diseases
I26-I28	Pulmonary heart disease and diseases of pulmonary circulation
I30-I52, except I46.9	Other forms of heart disease, excluding I46.9 (cardiac arrest, unspecified)
I60-I69	Cerebrovascular diseases
I70-I79, excluding I78 and I79	Diseases of arteries, arterioles and capillaries, excluding I78 (diseases of capillaries) and I79 (disorders of arteries, arterioles and capillaries in diseases classified elsewhere)

**Note:** This listing of diagnosis codes excludes: (a) Diseases of veins, lymphatic vessels and lymph nodes, not elsewhere classified (I80-I89) and (b) Other and unspecified disorders of the circulatory system (I95-I99)

In vital statistics registrations, which were used to validate the algorithm, cardiovascular deaths were those that had an underlying cause of death with a cardiovascular disease diagnosis. The relevant ICD-10 codes are provided in Table 2.

### Statistical analysis

The validation cohort was described using frequencies and percentages. Validity of the cardiovascular mortality algorithm was assessed using sensitivity, specificity, positive predictive value (PPV) and negative predictive value (NPV). All estimates are reported as percentages.

Sensitivity was calculated as the number of correctly-identified cardiovascular deaths in administrative health records divided by the total number of cardiovascular deaths from vital statistics registrations. Specificity was calculated as the number of correctly-identified non-cardiovascular deaths from administrative health records divided by the total number of non-cardiovascular deaths from vital statistics registrations. PPV was calculated as the number of correctly-identified cardiovascular deaths in administrative health records divided by the total number of cardiovascular deaths identified from administrative health records. NPV was calculated as the number of correctly-identified non-cardiovascular deaths in administrative health records divided by the total number of non-cardiovascular deaths identified from administrative health records. The 95% confidence intervals (CIs) were calculated for all estimates; they were based on the binomial distribution.

Estimates were produced overall (i.e., by combining frequencies for the six sites and then calculating the validity estimates), for the five Canadian provinces, and individually for each of the six sites. Overall and site-specific estimates were also stratified by location of death (in-hospital; out-of-hospital), sex and age group (< 70 years; ≥70 years).

## Results

**Fig. 1:**
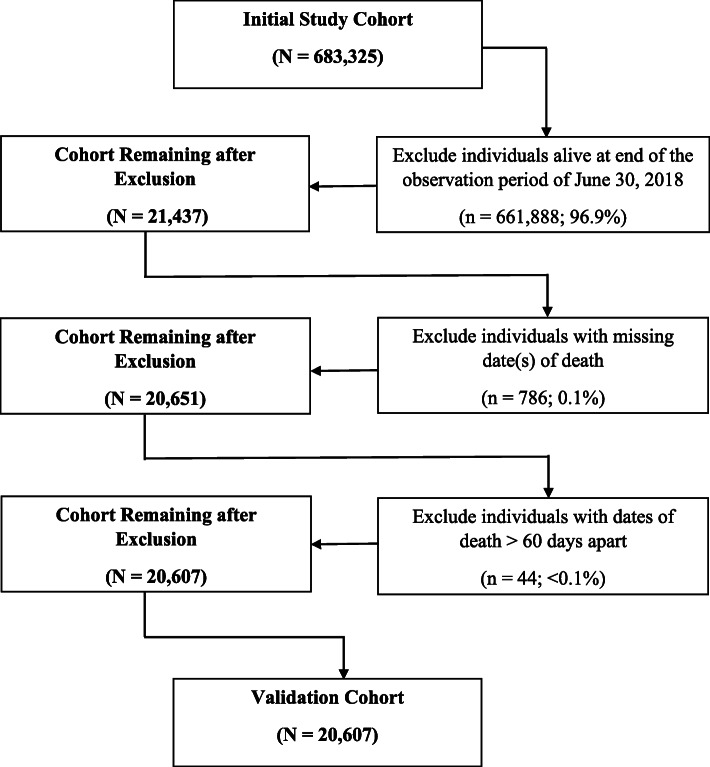
Study flow chart for development of the validation cohort. Legend: Initial study cohort was from an existing multi-database retrospective cohort study about the safety and effectiveness of sodium-glucose cotransporter-2 (SGLT2) inhibitors compared to dipeptidyl peptidase-4 (DPP4) inhibitors

As Fig. 1 reveals, the initial study cohort was comprised of 683,325 individuals of whom 96.9% were alive on June 30, 2018. There were few additional exclusions to arrive at the final validation cohort of 20,607 individuals. Specifically, less than 0.1% of individuals were missing a date of death in at least one data source or had dates of death greater than 60 days apart in administrative health records and vital statistics registrations.

More than two-thirds of the validation cohort (Table 3) were from the Canadian provinces of Ontario and Quebec. More than half (58.3%) of the validation cohort members were male and more than three-quarters were at least 70 years of age. The majority of validation cohort members had dates of death in 2016 or 2017 (data not shown). Overall, 31.7% of the deaths captured in vital statistics registrations were cardiovascular deaths.

**Table 3:** Characteristics of the validation cohort

Characteristic	All Sites N (%)	Canadian Sites						CPRD N (%)
		All Canadian Sites N (%)	AB N (%)	BC N (%)	MB N (%)	ON N (%)	QC N (%)	
Total	20,607 (100.0)	19,324 (93.8)	1125 (5.5)	3415 (16.6)	773 (3.8)	7741 (37.6)	6270 (30.4)	1283 (6.2)
Sex								
Female	8586 (41.7)	8056 (41.7)	403 (35.8)	1275 (37.3)	325 (42.0)	3421 (44.2)	2632 (42.0)	530 (41.3)
Male	12,021 (58.3)	11,268 (58.3)	722 (64.2)	2140 (62.7)	448 (58.0)	4320 (55.8)	3638 (58.0)	753 (58.7)
Age Group								
< 70 years	4702 (22.8)	4380 (22.7)	494 (43.9)	942 (27.6)	345 (44.6)	889 (11.5)	1710 (27.3)	322 (25.1)
≥ 70 years	15,905 (77.2)	14,944 (77.3)	631 (56.1)	2473 (72.4)	428 (55.4)	6852 (88.5)	4560 (72.7)	961 (74.9)
All Cardiovascular Deaths and Non-Cardiovascular Deaths								
Cardiovascular deaths identified from both sources	4236 (20.6)	3879 (20.1)	319 (28.4)	461 (13.5)	230 (29.8)	1795 (23.2)	1074 (17.1)	357 (27.8)
Cardiovascular deaths identified from administrative health records only	3537 (17.2)	3128 (16.2)	241 (21.4)	893 (26.1)	86 (11.1)	1119 (14.5)	789 (12.6)	409 (31.9)
Cardiovascular deaths identified from vital statistics registrations only	2300 (11.2)	2248 (11.6)	65 (5.8)	256 (7.5)	190 (24.6)	1034 (13.4)	703 (11.2)	52 (4.1)
Non-cardiovascular deaths identified from both sources	10,534 (51.1)	10,069 (52.1)	500 (44.4)	1805 (52.9)	267 (34.5)	3793 (49.0)	3704 (59.1)	465 (36.2)
In-Hospital Cardiovascular Deaths and Non-Cardiovascular Deaths^a^								
Cardiovascular deaths identified from both sources	1839 (17.0)	1687 (16.5)	121 (19.4)	227 (13.7)	78 (16.3)	694 (18.5)	567 (15.3)	152 (25.6)
Cardiovascular deaths identified from administrative health records only	848 (7.8)	635 (6.2)	84 (13.4)	162 (9.8)	13 (2.7)	124 (3.3)	252 (6.8)	213 (35.9)
Cardiovascular deaths identified from vital statistics registrations only	1383 (12.8)	1360 (13.3)	45 (7.2)	177 (10.7)	157 (32.8)	613 (16.4)	368 (9.9)	23 (3.9)
Non-cardiovascular deaths identified from both sources	6737 (62.3)	6531 (63.9)	375 (60.0)	1089 (65.8)	230 (48.1)	2313 (61.8)	2524 (68.0)	206 (34.7)
Total number of in-hospital deaths identified from both sources	10,807	10,213	625	1655	478	3744	3711	594
Out-of-Hospital Cardiovascular Deaths and Non-Cardiovascular Deaths^b^								
Cardiovascular deaths identified from both sources	2397 (24.5)	2192 (24.1)	198 (39.6)	234 (13.3)	152 (51.5)	1101 (27.5)	507 (19.8)	205 (29.8)
Cardiovascular deaths identified from administrative health records only	2689 (27.4)	2493 (27.4)	157 (31.4)	731 (41.5)	73 (24.7)	995 (24.9)	537 (21.0)	196 (28.4)
Cardiovascular deaths identified from vital statistics registrations only	917 (9.4)	888 (9.7)	20 (4.0)	79 (4.5)	33 (11.2)	421 (10.5)	335 (13.1)	29 (4.2)
Non-cardiovascular deaths identified from both sources	3797 (38.7)	3538 (38.8)	125 (25.0)	716 (40.7)	37 (12.5)	1480 (37.0)	1180 (46.1)	259 (37.6)
Total number of out-of-hospital deaths identified from both sources	9800	9111	500	1760	295	3997	2559	689

**Note:** Percentages may not sum to 100% due to rounding. *AB* Alberta, *BC* British Columbia, *MB* Manitoba, *ON* Ontario, *QC* Quebec, *CPRD* UK Clinical Practice Research Datalink. Classification of a death as in-hospital or out-of-hospital was made using administrative health data

^a^Percentages are based on total number of in-hospital deaths

^b^Percentages are based on total number of out-of-hospital deaths

Slightly more than half (10,807; 52.4%) of the deaths included in the validation study were identified as in-hospital deaths (Table 3). Amongst all in-hospital deaths, 17.8% were identified as cardiovascular deaths in both data sources and 62.3% were identified as non-cardiovascular deaths in both data sources. Amongst all out-of-hospital deaths, 24.5% were identified as cardiovascular deaths in both data sources and 38.7% were identified as non-cardiovascular deaths in both data sources.

Table 4 contains the validity estimates for the cardiovascular mortality algorithm for individual sites, as well as overall and for the Canadian provinces. Overall estimates were 64.8% (95% CI 63.6, 66.0) for sensitivity, 74.9% (95% CI 74.1, 75.6) for specificity, 54.5% (95% CI 53.7, 55.3) for PPV, and 82.1% (95% CI 81.6, 82.6) for NPV.

**Table 4:** Validity estimates (95% confidence intervals) for the cardiovascular mortality algorithm, overall and by site

Measure	All Sites	Canadian Sites						CPRD
		All Canadian Sites	AB	BC	MB	ON	QC	
Sensitivity	64.8 (63.6, 66.0)	63.3 (62.1, 64.5)	83.1 (78.9, 86.6)	64.3 (60.6, 67.7)	54.8 (49.9, 59.6)	63.4 (61.6, 65.2)	60.4 (58.1, 62.7)	*87.3* (83.6, 90.3)
Specificity	74.9 (74.1, 75.6)	76.3 (75.6, 77.0)	67.5 (64.0, 70.8)	66.9 (65.0, 68.6)	75.6 (70.8, 80.0)	77.2 (76.0, 78.4)	*82.4* (81.5, 83.7)	53.2 (49.8, 56.5)
PPV	54.5 (53.7, 55.3)	55.4 (54.5, 56.3)	57.0 (52.7, 61.1)	34.0 (31.4, 36.5)	*72.8* (67.5, 77.5)	61.6 (60.2, 63.0)	57.6 (55.6, 60.2)	46.6 (43.0, 50.2)
NPV	82.1 (81.6, 82.6)	81.7 (81.2, 82.3)	88.5 (85.5, 90.9)	87.6 (86.1, 89.0)	58.4 (53.7, 63.0)	78.6 (77.7, 79.4)	84.0 (82.9, 85.1)	*89.9* (86.9, 92.3)

**Note:** All estimates are expressed as %. PPV = positive predictive value, NPV = negative predictive value, *AB* Alberta, *BC* British Columbia, *MB* Manitoba, *ON* Ontario, *QC* Quebec, *CPRD* UK Clinical Practice Research Datalink. Highest and lowest site-specific estimates for each measure are delineated by italics and underline font, respectively

The CPRD produced the highest site-specific estimates of overall sensitivity (87.3%; 95% CI 83.6, 90.3) and NPV (89.9%; 95% CI 86.9, 92.3). The Canadian province of Manitoba produced the lowest estimates of sensitivity (54.8%; 95% CI 49.9, 59.6) and NPV (58.3%; 95% CI 53.7, 63.0), but the highest estimate of PPV (72.8%; 95% CI 67.5, 77.5). The province of Quebec had the highest estimate of specificity (82.8%; 95% CI 81.5, 83.7). The lowest estimate of PPV was for the province of British Columbia (33.9%; 95% CI 31.4, 36.5).

**Fig. 2:**
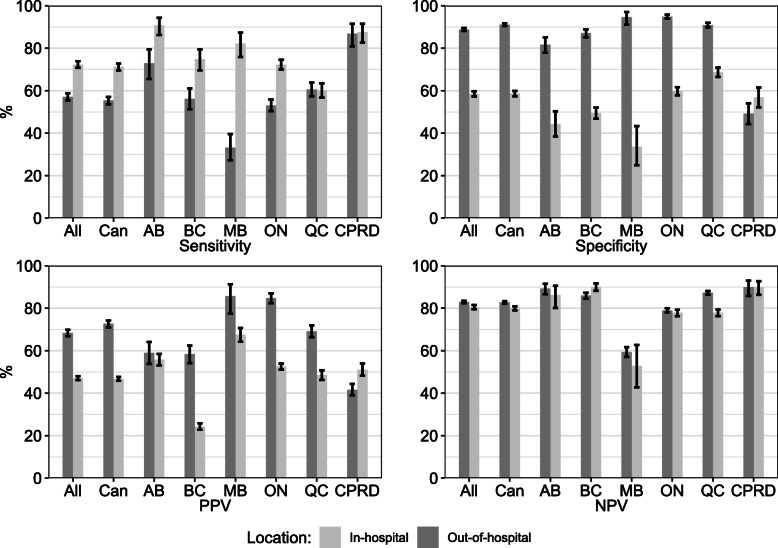
Validity estimates (%) for the cardiovascular mortality algorithm, by location of death. Legend: Error bars = 95% confidence intervals, All = all sites, Can = all Canadian sites, PPV = positive predictive value, NPV = negative predictive value, AB = Alberta, BC = British Columbia, MB = Manitoba, ON = Ontario, QC = Quebec, CPRD = UK Clinical Practice Research Datalink

Figure 2 provides validity estimates for the cardiovascular mortality algorithm stratified by location of death (i.e., in-hospital versus out-of-hospital). Overall sensitivity was 57.1% (95% CI 55.4, 58.8) for in-hospital deaths and 72.3% (95% CI 70.8, 73.9) for out-of-hospital deaths. Overall specificity was 88.8% (95% CI 88.1, 89.5) for in-hospital deaths and 58.5% (95% CI 57.3, 59.7) for out-of-hospital deaths. Overall PPV was 68.4% (95% CI 66.9, 69.9) for in-hospital deaths and 47.1% (95% CI 46.2, 48.0) for out-of-hospital deaths. Overall NPV was similar in both locations (83.0% for in-hospital, 95% CI 82.4, 83.5; 80.5% for out-of-hospital, 95% CI 79.6, 81.5). Sensitivity was higher for out-of-hospital deaths than for in-hospital deaths in all sites with the exception of Quebec and the CPRD. Specificity and PPV were higher for all sites for in-hospital deaths, with the exception of the CPRD.

Validity estimates were stratified by sex and age group, respectively (see Additional File 1). Sensitivity estimates were similar at all sites for males and females and for younger and older age groups. Specificity estimates were similar at all sites, except for Ontario where the estimates were lower for males than females and for older than younger cohort members. The same was true for PPV, which was lower for older than younger age groups in Ontario. The PPV estimate for the Canadian province of Alberta was lower for females than males. Estimates of NPV were similar across all sites.

## Discussion

In this study, we applied an algorithm to administrative health records to identify cardiovascular deaths. We assessed the validity of this algorithm using vital statistics registrations, which contain information about the underlying cause of death. The study was conducted using data from five Canadian provinces and the UK. Overall validity estimates were modest, suggesting that the algorithm had moderate to low validity for identifying cardiovascular deaths. However, there was substantial variability across study sites.

The cardiovascular algorithm resulted in slightly less than one-half of the cardiovascular deaths being identified as out-of-hospital deaths; a US study found about one-third of cardiovascular deaths occurred out of hospital [24], although these results were based only on ischemic heart disease deaths and were for an earlier time period (1979 to 1989) than our study observation period; a Swedish study reported an increasing rate of out-of-hospital cardiovascular deaths between 1991 and 2006 [22]. Not unexpectedly, the algorithm had greater specificity and PPV but lower sensitivity for in-hospital deaths than for out-of-hospital death for most sites due to the challenges of identifying the specific cause for out-of-hospital deaths.

Variation in the validation results are consistent with the results of a previous multi-database study conducted by CNODES that showed substantial variation across Canadian provinces in the association of medication exposure with health outcomes [26]; these variations were attributed to differences in the data, including diagnostic coding practices. While Canada has a universal healthcare system, the responsibility for delivery of services exists with the individual provinces and territories. A consequence is that administrative health records are not captured in a standardized way in all jurisdictions with the exception of hospitalization data, which are standardized in all provinces except Quebec. As well, the training and skills of coders across jurisdictions is unlikely to be the same, because there are no national standards for this training. Examination of our site-specific validation results revealed that the CPRD data from the UK had the highest overall sensitivity and NPV. This finding might be attributed to differences in the data (i.e., primary care electronic medical records versus physician billing claims), coding practices, and/or differences in healthcare use (e.g., likelihood of hospitalization) between the UK and Canada.

In addition to conducting this validation study, we compared the risk estimates obtained using the cardiovascular mortality algorithm and the risk estimates obtained using cardiovascular deaths from vital statistics registrations in a real-world study about SGLT2 inhibitors compared to DPP4 inhibitors [14]. A composite endpoint of major adverse cardiovascular events (MACE) was constructed, which included myocardial infarction, ischaemic stroke, and cardiovascular death. When the composite endpoint used the cardiovascular mortality algorithm to identify cardiovascular deaths, a hazards ratio (HR) of 0.76 (95% CI 0.69, 0.84) was produced for SGLT2 inhibitors compared to DPP4 inhibitors (number of events: 2146 for SGLT2 inhibitors; 3001 for DPP4 inhibitors). When the composite endpoint used vital statistics registrations to identify cardiovascular deaths, the HR was similar (0.78; 95% CI 0.63, 0.97) for SGLT2 inhibitors compared to DPP4 inhibitors (number of events: 920 for SGLT2 inhibitors; 1257 for DPP4 inhibitors).

A major strength of this study is the assessment of validity of the cardiovascular mortality algorithm across multiple sites, including both Canadian and UK sites with different healthcare systems and healthcare use. Within Canada, the vast majority of validation studies for administrative health data algorithms have only been conducted in a single site [27], which limits their potential generalizability. Another strength is that we examined validity of an algorithm for a commonly-used endpoint in drug safety studies. Finally, we produced site-specific estimates of sensitivity, specificity, PPV, and NPV so that the magnitude of potential misclassification bias can be assessed at the site level.

This study is not without limitations. First, we acknowledge that vital statistics registrations may not be error free. Statistics Canada notes that the last comprehensive investigation of errors in vital statistics registrations occurred in the 1980s, although some province-specific data quality assessments have since been conducted [3]. Errors in the cause of death recorded in the vital statistics registrations, which could result in bias and loss of precision in the validity estimates, may arise because of differences of interpretation amongst coders about the information contained on a death certificate [28]. One US study found that for coronary heart disease deaths, death certificates had sensitivity of 84%, PPV of 67%, specificity of 84%, and NPV of 93% when a physician panel assessment of cause of death was adopted as the reference standard [29]. A multi-site US study of coronary heart disease deaths in death certificates reported PPV of 67% and sensitivity of 81% when physician review of cause of death was used as the reference standard; there was substantial variation across sites in these estimates, as well as for in-hospital versus out-of-hospital deaths [30]. The authors of this study also noted the challenges associated with classifying a death as a coronary heart disease death versus a non-coronary heart disease death using diagnosis codes. As well, we acknowledge that the results of this study may not generalize to the population of each jurisdiction because the original study cohort was limited to individuals receiving selected antidiabetic medications and the majority (i.e., greater than 75%) were at least 70 years of age. A recent review paper reported that the cardiovascular death rate amongst individuals with diabetes was approximately 4.5 times greater than amongst individuals without diabetes of the same age, without considering other cardiovascular risk factors [31]. Our estimates of PPV and NPV may not generalize because they are influenced by prevalence of cardiovascular disease in the population; as prevalence increases, PPV will also increase but NPV will decrease [32]. Older populations under treatment for diabetes have more underlying comorbid conditions and therefore are a more challenging group in which to identify the underlying cause of death than the general population [28], which could result in misclassification of cause of death.

Future research could validate the proposed cardiovascular mortality algorithm in a general population as opposed to a treatment-specific population. As well, a model-based approach could be explored as an alternative approach to develop an algorithm for cardiovascular mortality. Machine-learning models that take account of multiple characteristics of the individual, including their history of comorbid conditions (e.g., hypertension, prior coronary artery disease) and relevant medications may result in increased accuracy. This finding of increased accuracy has been observed for cardiovascular disease risk predictions from machine-learning algorithms when compared to risk predictions based on conventional statistical models [33].

## Conclusions

Cardiovascular diseases are a major cause of death worldwide. A cardiovascular mortality algorithm based on routinely-collected administrative health records is therefore potentially valuable for many population-based studies, including those about comparative effectiveness of new healthcare interventions or treatments, such as new prescription medications. This study found only modest overall validity of the cardiovascular mortality algorithm when compared with vital statistics registrations, but substantial variation in validity estimates across sites. This variation suggests there are opportunities for methodological studies to address the bias associated with using a cardiovascular mortality algorithm derived from administrative health records.

## Supplementary Information

**Additional file 1.** Validity estimates for a cardiovascular mortality algorithm applied to administrative health records stratified by sex and age group. This file contains estimates of sensitivity, specificity, positive predictive value, and negative predictive value stratified by sex and age group (< 70 years; ≥70 years).

## Data Availability

The data that support the findings of this study are not publicly available, in accordance with site-specific privacy restrictions. The data that support the findings of this study are available, with submission of appropriate ethics and data access approvals, from Alberta Health, the British Columbia Ministry of Health, the Manitoba Centre for Health Policy, the Institute for Clinical Evaluative Sciences (ICES), the *Institut national d’excellence en santé et en services sociaux (INESSS)*, and the Independent Scientific Advisory Committee (ISAC) of the CPRD .

## Abbreviations

CI: Confidence interval
CNODES: Canadian Network for Observational Drug Effect Studies
CPRD: Clinical Practice Research Datalink
DPP-4: Dipeptidyl peptidase-4
ED: Emergency department
HES: Hospital episode statistics
HR: Hazard ratio
ICD: International Statistical Classification of Diseases and Related Problems
MACE: Major adverse cardiovascular events
NPV: Negative predictive value
ONS: Office of National Statistics
PPV: Positive predictive value
SGLT2: Sodium-glucose cotransporter-2
UK: United Kingdom
