# Supplementary material for: Validity of an algorithm to identify cardiovascular deaths from administrative health records: a multi-database population-based cohort study
Source: BMC Health Serv Res. 2021 Jul 31;21:758. doi: 10.1186/s12913-021-06762-0 (PMC8325284; doi:10.1186/s12913-021-06762-0)

**Validity of an Algorithm to Identify Cardiovascular Deaths from Administrative Health Records: A Multi-Database Population-Based Cohort Study**

**Lisa M. Lix PhD, Shamsia Sobhan MSc, Audray St-Jean MSc, Jean-Marc Daigle MSc, Anat Fisher MD PhD, Oriana H.Y. Yu MD MSc, Sophie Dell'Aniello MSc, Nianping Hu PhD, Shawn C. Bugden PharmD MSc, Baiju R. Shah MD PhD, Paul E. Ronksley PhD, Silvia Alessi-Severini PhD, Antonios Douros MD PhD, Pierre Ernst MD MSc, Kristian B. Filion PhD**

**Additional File 1: Validity estimates for a cardiovascular mortality algorithm applied to administrative health records stratified by sex and age group**

**Additional Figure 1: Validity estimates (%) for a cardiovascular mortality algorithm, by sex**

**Figure legend:** Error bars = 95% confidence intervals; All = all sites; Can = all Canadian sites; PPV = positive predictive value; NPV = negative predictive value; AB = Alberta; BC = British Columbia; MB = Manitoba; ON = Ontario; QC = Quebec; CPRD = UK Clinical Practice Research Datalink

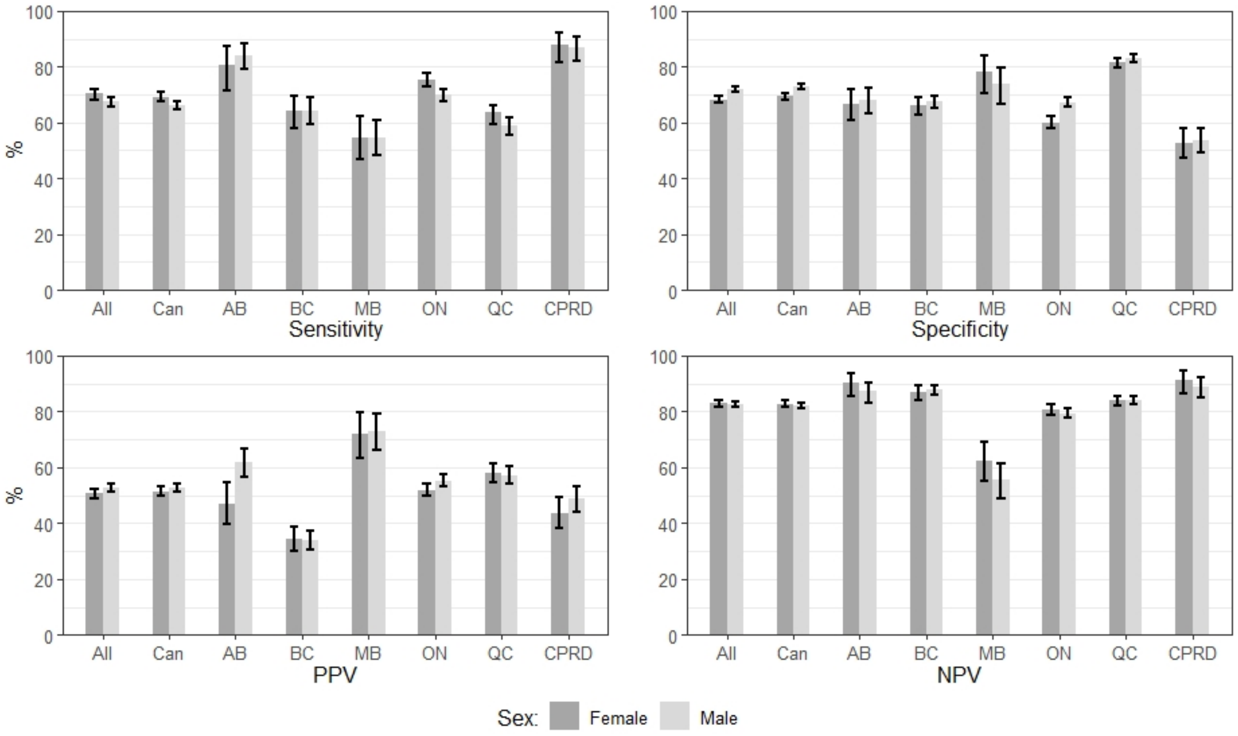

**Additional Figure 2: Validity estimates (%) for a cardiovascular mortality algorithm, by age group**

**Figure legend:** Error bars = 95% confidence intervals; All = all sites; Can = all Canadian sites; PPV = positive predictive value; NPV = negative predictive value; AB = Alberta; BC = British Columbia; MB = Manitoba; ON = Ontario; QC = Quebec; CPRD = UK Clinical Practice Research Datalink

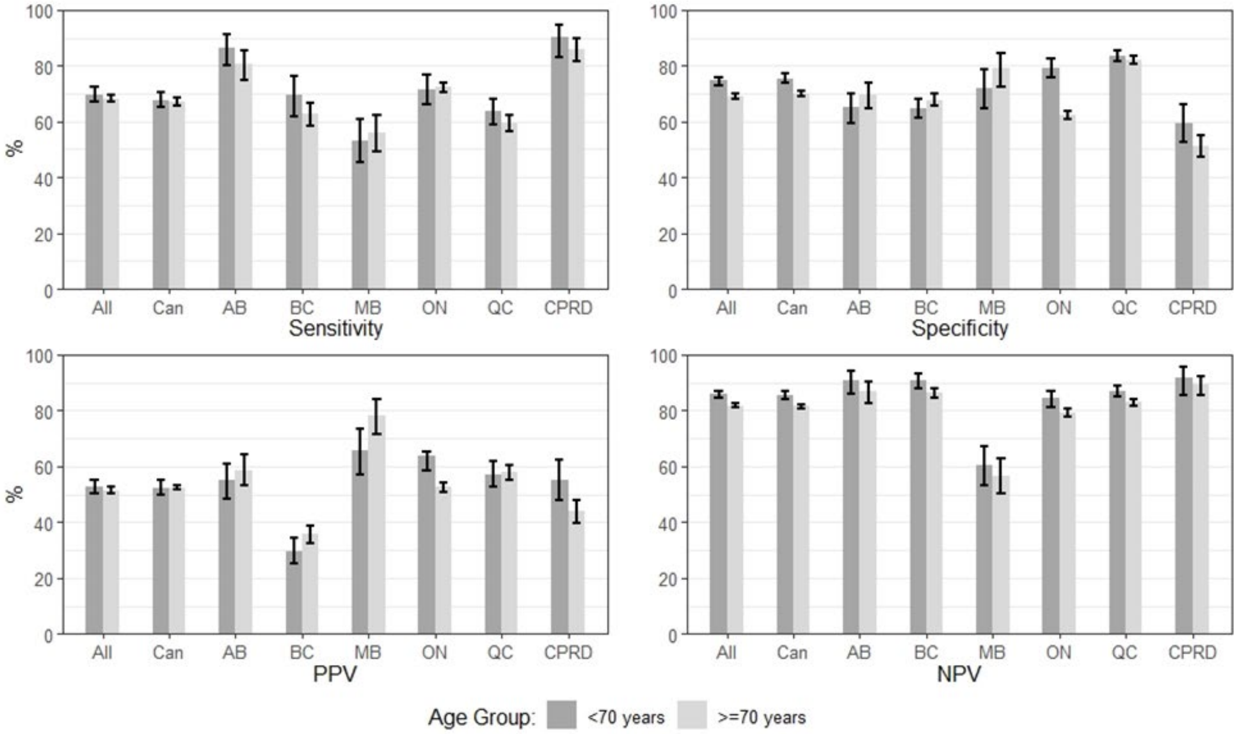

Supplement: Supplementary file 1 — Additional file 1. Validity estimates for a cardiovascular mortality algorithm applied to administrative health records stratified by sex and age group. This file contains estimates of sensitivity, specificity, positive predictive value, and negative predictive value stratified by sex and age group (< 70 years; ≥70 years). [file 12913_2021_6762_MOESM1_ESM.pdf]
